# Supplementary material for: Genetic regulation of fasting-induced longevity effects
Source: Genetics. 2026 Feb 17;233(1):iyag045. doi: 10.1093/genetics/iyag045 (PMC13147523; doi:10.1093/genetics/iyag045)
Supplement: iyag045_Supplementary_Data [file iyag045_supplementary_data.zip › Supplemental_Material_GENETICS-2025-308551.pdf]

Supplemental Materials for ‘Genetic regulation of fasting-induced longevity effects’

Alison Luciano[1], Laura Robinson[1], William H. Schott[1], A. Phillip West[1], Ron Korstanje[1], Gary A. Churchill [1,*]
[1]The Jackson Laboratory, Bar Harbor, ME, 04609, USA
[*]Correspondence: gary.churchill@jax.org

Supplementary Figure S1: Sex-specific effects of dietary intervention on lifespan across genetically diverse mouse strains. A) Kaplan-Meier curves and interaction contrasts for lifespan response to diet among males (dashed) and females (solid) reveal inter-strain variation in IF response. Abbreviations: mo. = months, int = interaction. B) Data from the CC Longitudinal Study show sexual dimorphism and genetic effects in IF response. Visualization highlights the interaction between sex, diet, and genetic background in modulating longevity. Lifespan is plotted for male and female mice, grouped by dietary regimen and genetic strain. Data are shown for ad libitum feeding (○) and 2-day intermittent fasting (△) and plotted by sex as median ± IQR.

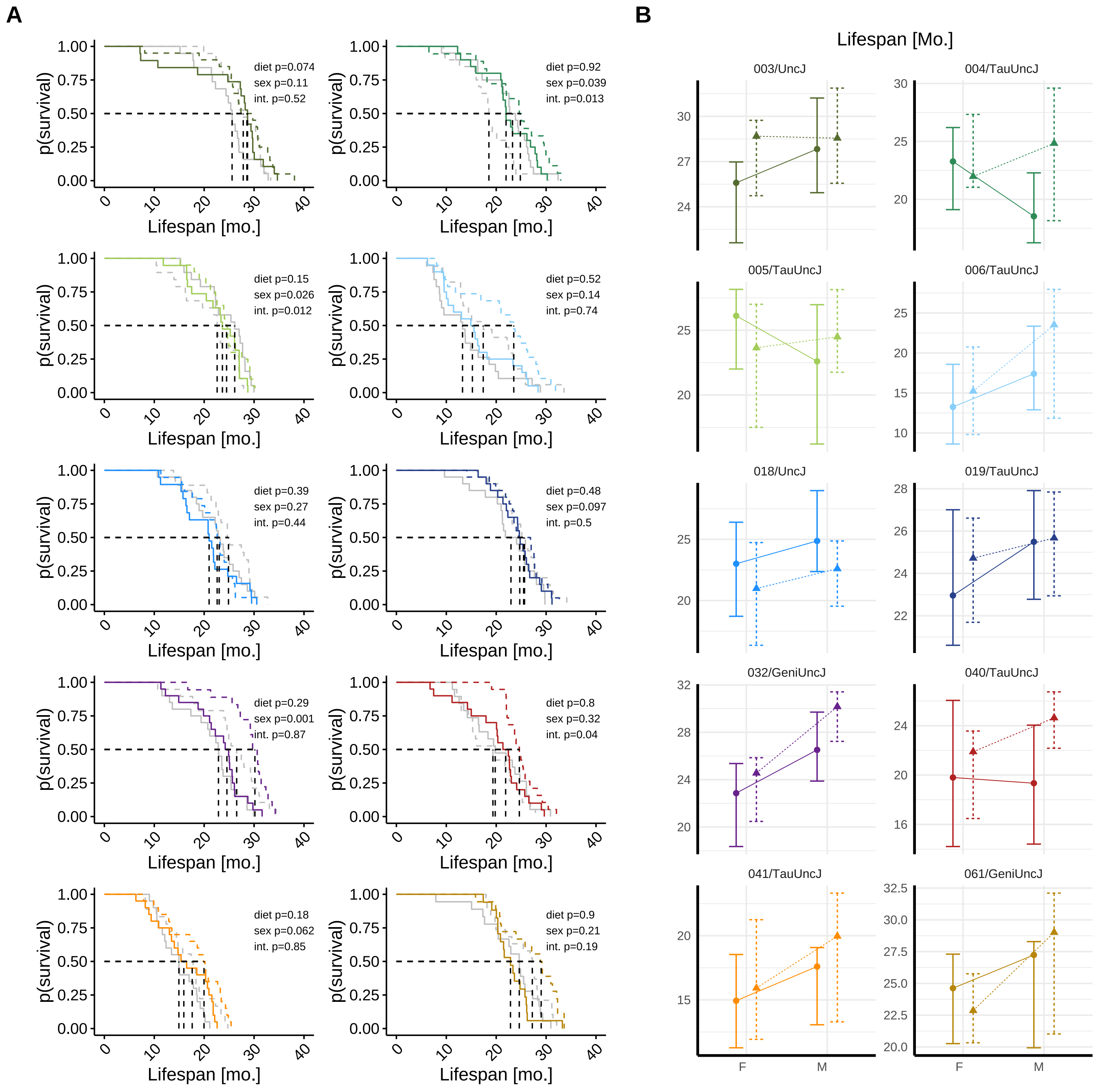

Supplementary Figure S2: Follow-up by physiologic trait, year, sex, and intervention group. For phenotyping domains assessed multiple times per year, e.g., frailty, any measurement within the study year was counted as continued follow-up. In total, longitudinal phenotyping included over 66,000 body weight measurements, 2,000 frailty assessments, and 1,000 each of hematology, immunology, metabolic phenotype, and glucose tolerance assays. Abbreviations: Fasted.Gl=fasted glucose, AL=*ad libitum*, IF=intermittent fasting.

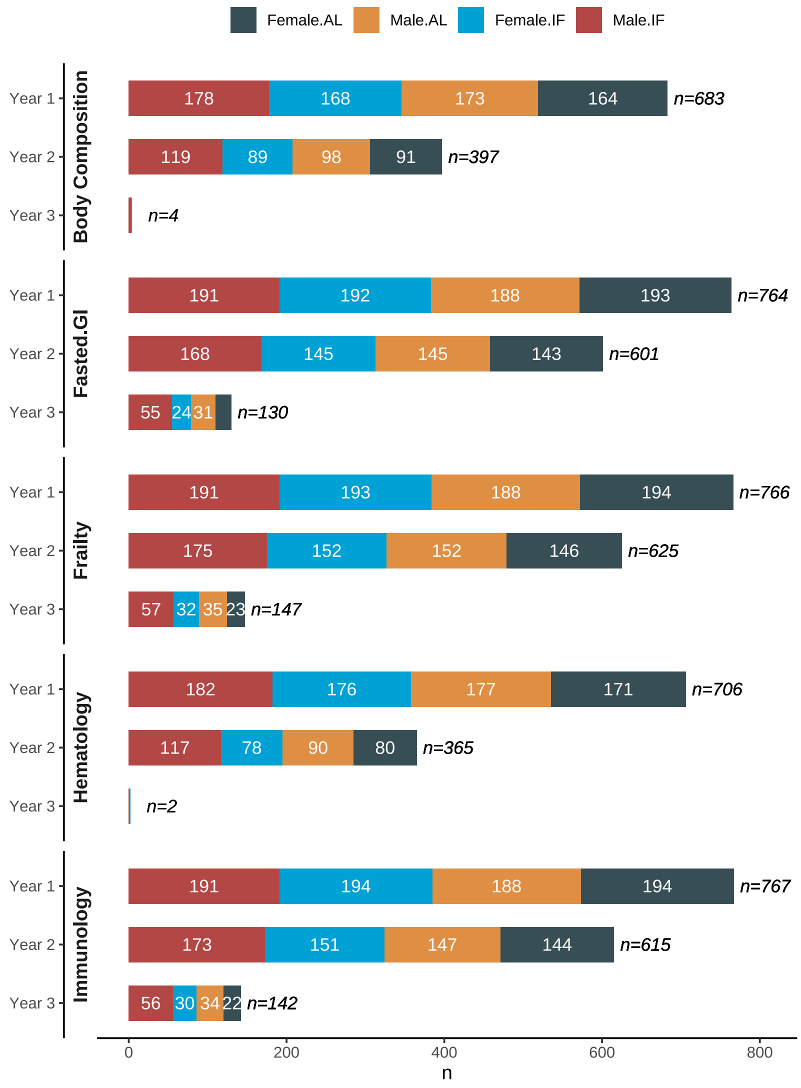

Supplementary Figure S3: Metabolic phenotype response to intermittent fasting (IF) is influenced by genetic background. Nuclear magnetic resonance (NMR) body composition assays were conducted at 10 and 22 months (n>1k). Month 22 NMR data were plotted as mean ± SE adiposity [%] (A) and lean mass [g] (B) for male and female mice, grouped by dietary regimen (IF [∆] or AL [○]) and genetic strain. Early lean mass loss in response to IF was observed with varied risk across strains and sexes. Some strains (e.g., 005/TauUncJ, 006/TauUncJ) showed minimal total mass change but significant lean mass loss, underscoring the importance of composition-specific metrics for characterizing metabolic phenotype. Plots for 10 month timepoint shown in **Fig. 3a-b**.

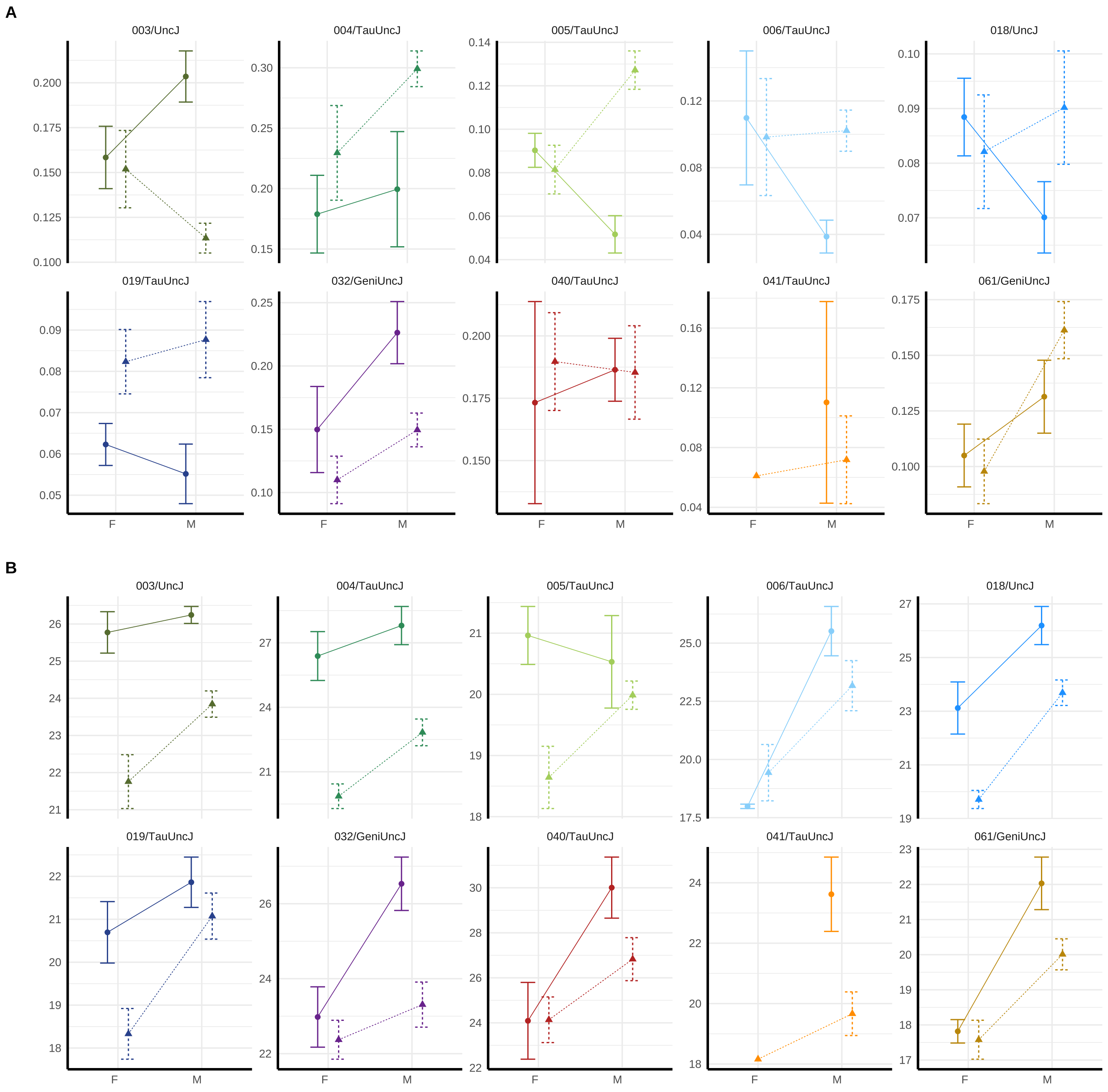

Supplementary Figure S4: Lifetime incidence of individual health deficits highlights several frailty indicators characterized by low susceptibility and minimal influence from genetic or dietary factors. Twenty-seven serially collected non-invasive biomarkers of frailty were measured at weeks 21 (preintervention), 43 (intervention onset), 95, 121, and 147 weeks (n>2,000). The comparison of cumulative incidence of individual health deficits across intervention groups (IF = color, AL = gray) reveals several physiologic systems with low aggregate risk of frailty accumulation across the lifespan regardless of genetic background or exposure to IF dietary regimen. Note: Remaining items shown in **Fig. 4**. Frailty index items were binarized as severe/nonsevere.

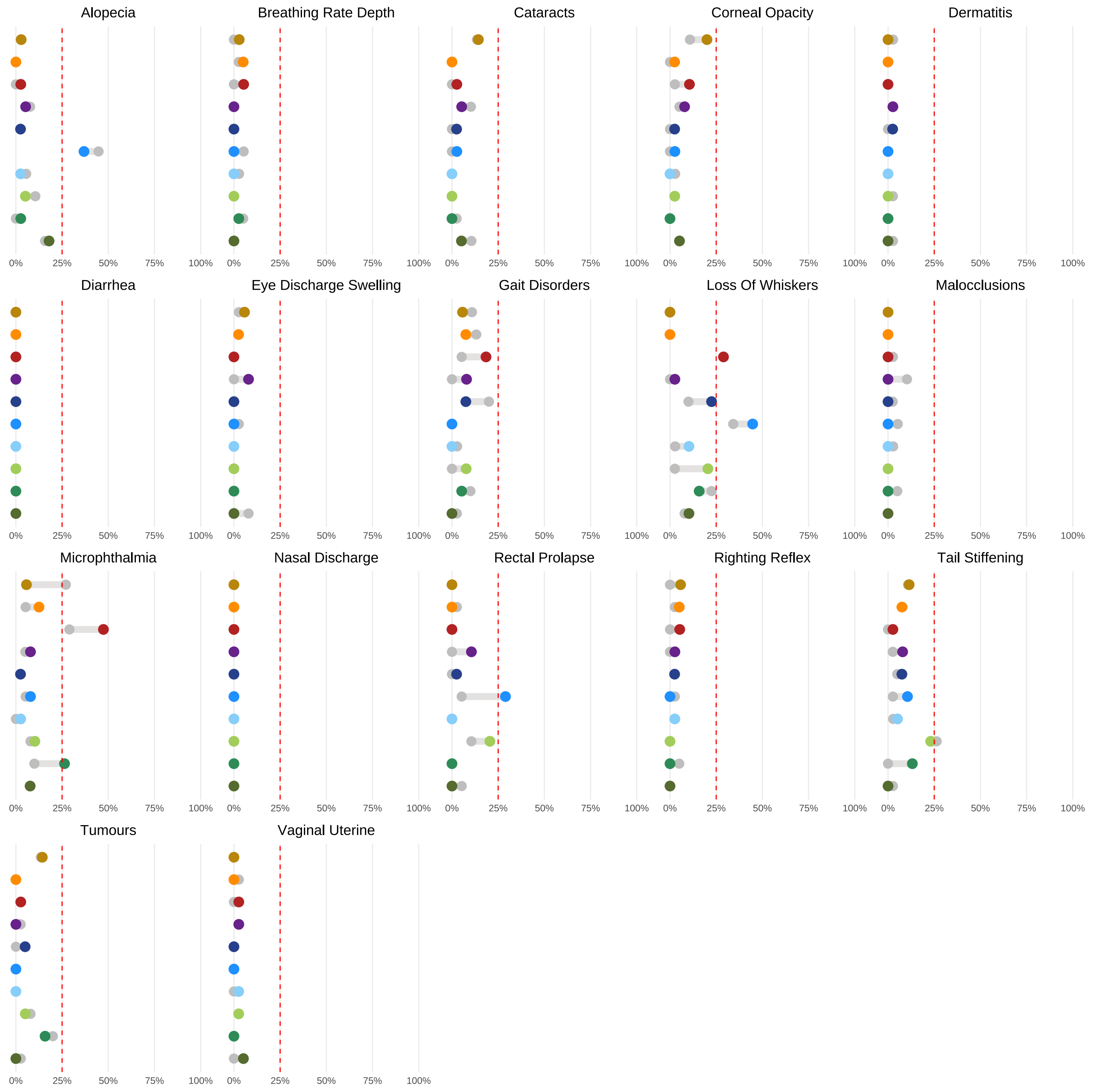

Supplementary Figure S5: Genetic signatures of hematologic response to IF in late life. Year 2 red blood cell distribution width (RDW CV) data (A) and mean corpuscular volume (MCV) (B) were plotted as mean ± SE for male and female mice grouped by dietary regimen (IF [Δ] or AL [○]) and genetic strain, demonstrating sexually dimorphic IF response at 22 months. Note: 10-month data for RDW and MCV are shown in **Fig. 5**.

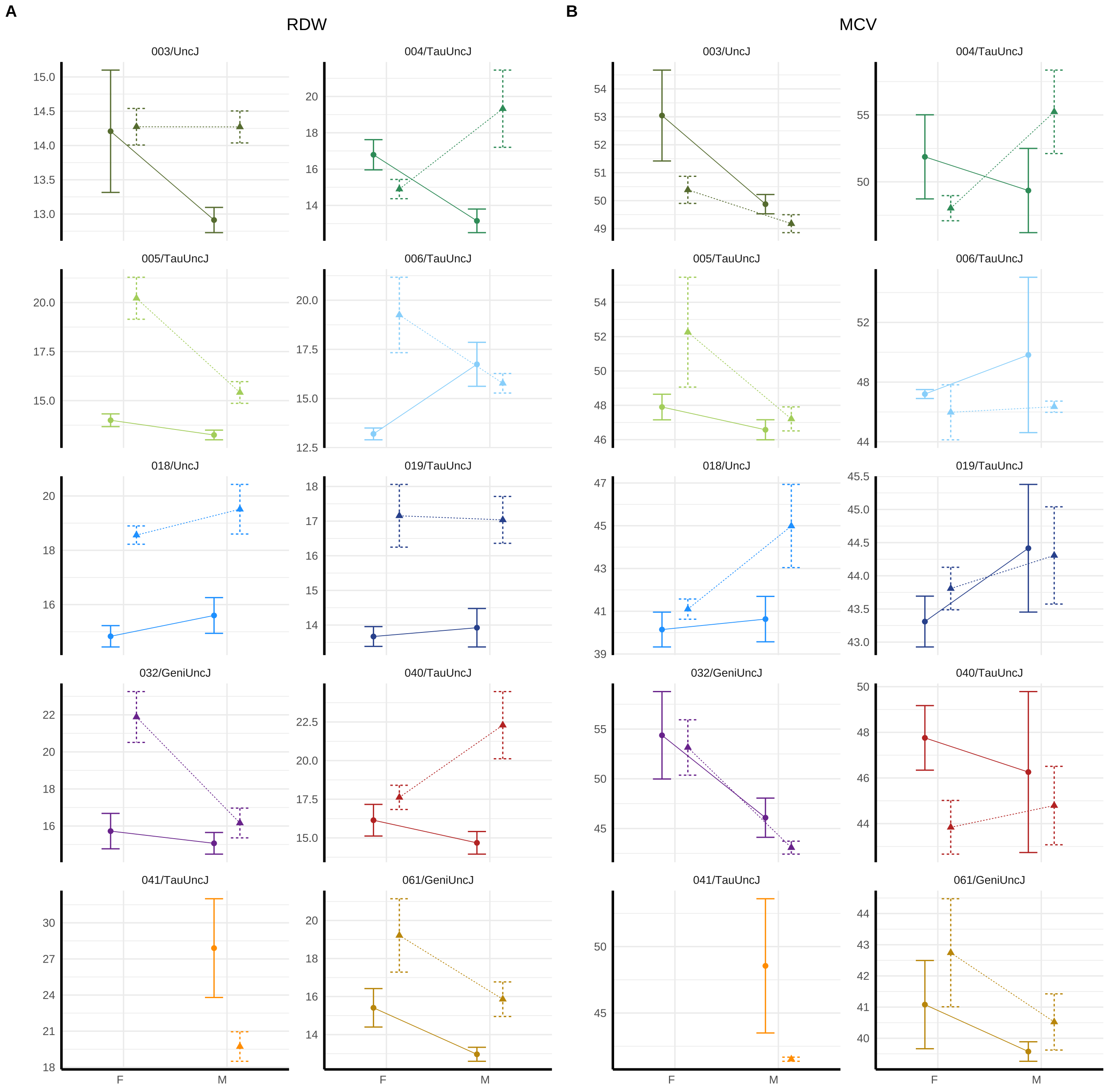

Supplementary Figure S6: Varying hematologic IF responses clustered by strain demonstrate how deep longitudinal phenotyping in the Collaborative Cross enables discovery of models for intervention response. Heatmaps compare diet effects by hematologic outcome for males and females at year 1 and year 2. Clustering partitions and dendrograms reveal latent groups of strains with similarly patterned hematologic intervention response. Notes: Row (hematologic trait) order for 45-week (year 1) female heatmap [top left; see row-wise dendrogram] determined by cluster analysis; row order for remaining plots were pre-specified without clustering to allow for visual comparison across study strata. Column (strain) order determined by clustering analysis for each heatmap separately with partitions to group strains with similar hematologic profile response to IF. The reference group for strain-specific diet effects shown in the heatmap is parameterized such that the diet comparison is IF-AL. Negative values indicate adjusted mean outcome for IF is lower than for the reference level AL. Abbreviations for hematologic traits are defined in Supplementary Table S6.

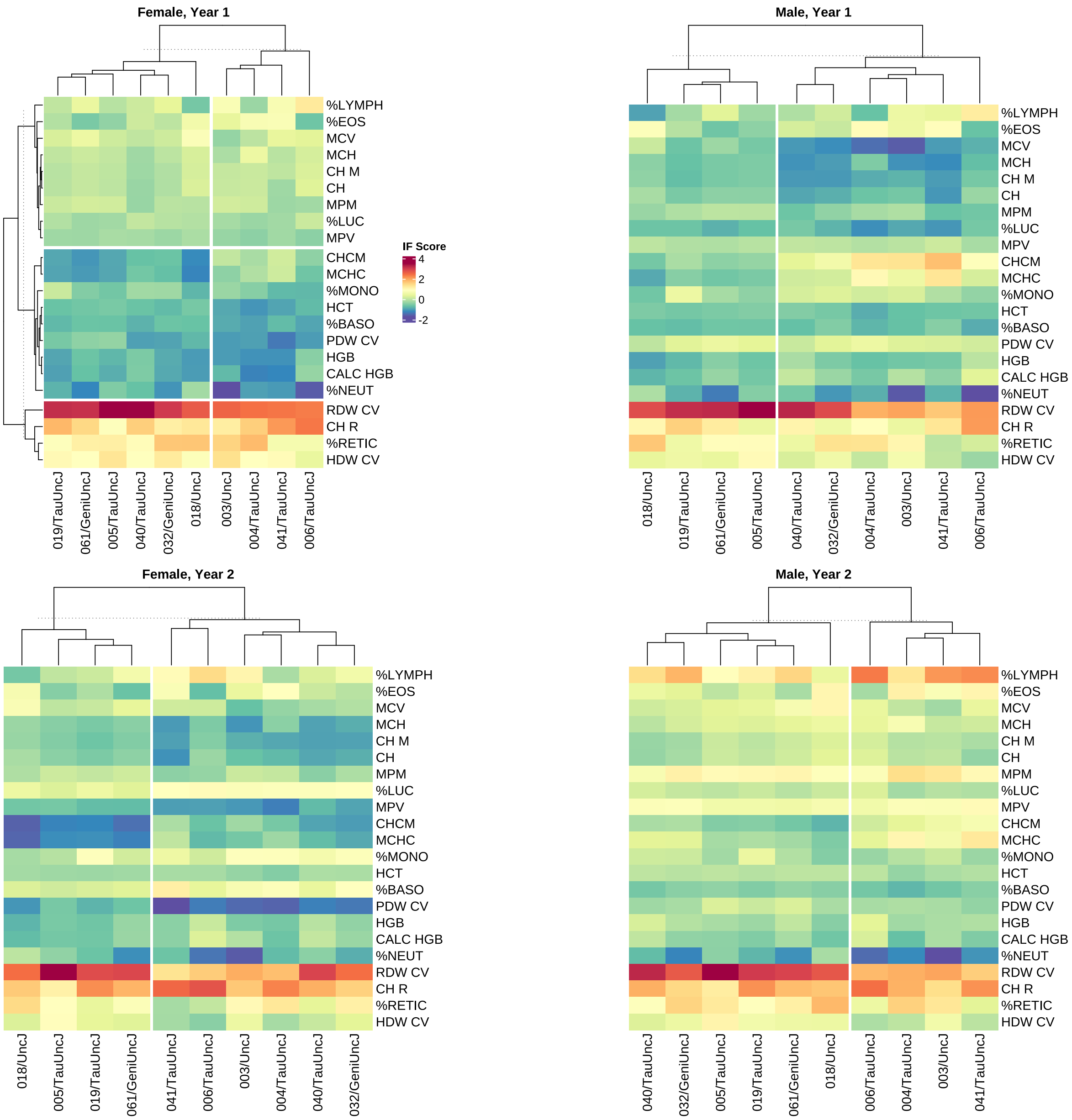

Supplementary Figure S7: Varying immunologic IF responses clustered by strain demonstrate how deep longitudinal phenotyping in the Collaborative Cross enables discovery of models for intervention response. Heatmaps compare diet effects by immunologic outcome for males and females at year 1 and year 2. Clustering partitions and dendrograms reveal latent groups of strains with similarly patterned immunologic intervention response. Notes: Row (immunologic trait) order for 45-week (year 1) female heatmap [top left; see row-wise dendrogram] determined by cluster analysis; row order for remaining plots were pre-specified without clustering to allow for visual comparison across study strata. Column (strain) order determined by clustering analysis for each heatmap separately with partitions to group strains with similar immunologic profile response to IF. The reference group for strain-specific diet effects shown in the heatmap is parameterized such that the diet comparison is IF-AL. Negative values indicate adjusted mean outcome for IF is lower than for the reference level AL. Abbreviations for immunologic traits are defined in Supplementary Table S6.

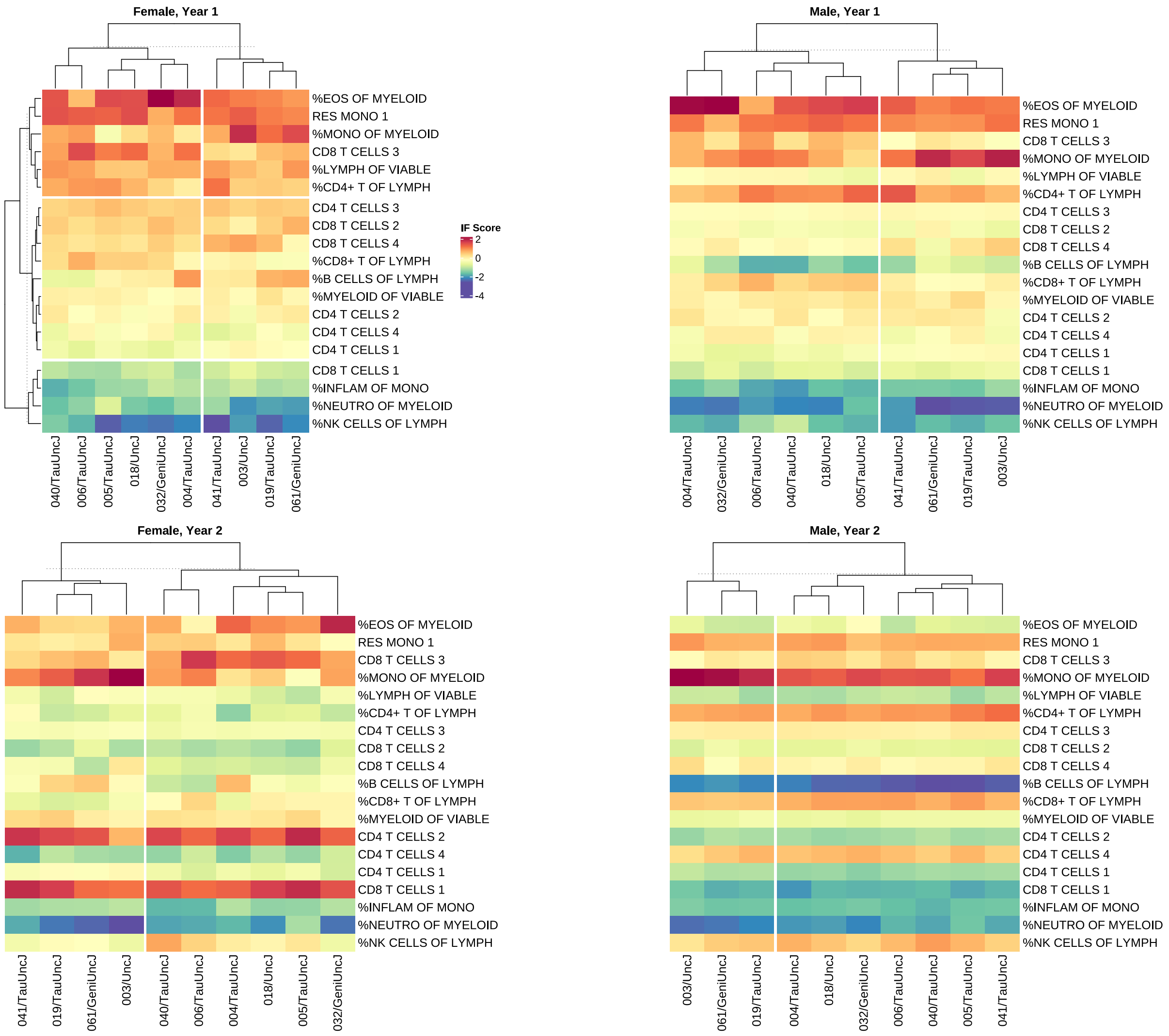

Supplementary Table S1: Comparison of diet effects on lifespan by sex.

(a) Median lifespan by diet and sex. Significance (p-value) of comparisons (1df) between diet groups from χ^2 tests.

	IF	AL	IF-AL	p
Female	21.97	22.24	-0.26	0.92
Males	24.84	23.17	1.66	0.02

(b) Maximum lifespan (90% survival) by diet and sex. Significance (p-value) of comparisons (1df) between diet groups from χ^2 tests.

	IF	AL	IF-AL	p
Females	29.05	28.59	0.46	0.49
Males	31.41	30.79	0.62	0.30

(c) Mean lifespan by diet and sex. Significance (p-value) of comparisons (1df) between diet groups from Restricted Mean Survival Time (RMST) analysis.

	IF	AL	IF-AL (95% CI)	p
Females	21.34	21.01	0.334 (-0.94, 1.61)	0.607
Males	24.37	22.35	2.02 (0.76, 3.28)	0.002

Supplementary Table S2: Descriptive statistics for lifespan. Survival time for specified quantiles were determined using the Kaplan-Meier estimator, with the desired percentile extracted via the quantile function applied to the survival curve. Maximum lifespan defined as 90th percentile lifespan within study strata. Values are rounded to the nearest 10th of a month.

(a) Lifespan summarized by strain and sex.

Strain	p50_F	p50_M	p25_F	p25_M	p75_F	p75_M	max_F	max_M
003/UncJ	26.9	27.8	22.3	25.4	29.6	31.2	32.0	33.3
004/TauUncJ	22.6	21.0	21.0	17.4	26.4	25.0	28.2	30.6
005/TauUncJ	25.3	23.9	20.4	19.7	27.2	27.0	28.8	29.1
006/TauUncJ	13.8	21.6	9.4	12.9	18.6	25.9	26.4	28.5
018/UncJ	21.9	23.4	17.0	21.2	25.8	26.2	29.4	29.3
019/TauUncJ	24.5	25.5	21.1	22.8	26.6	27.8	29.6	31.0
032/GeniUncJ	23.5	29.2	19.6	24.9	25.6	31.0	28.7	33.1
040/TauUncJ	21.3	22.6	15.0	19.1	24.7	25.3	26.7	28.2
041/TauUncJ	15.0	18.5	11.3	13.1	20.0	22.7	21.7	24.5
061/GeniUncJ	23.4	27.2	20.3	21.0	26.0	31.2	28.8	32.3

(b) Lifespan summarized by strain, sex, and diet.

Strain	Diet	p50_F	p50_M	p25_F	p25_M	p75_F	p75_M	max_F	max_M
003/UncJ	AL	25.6	27.8	21.6	24.9	27.0	31.2	32.0	32.3
003/UncJ	IF	28.7	28.6	24.7	25.6	29.7	31.9	34.0	33.9
004/TauUncJ	AL	23.3	18.5	19.1	16.2	26.2	22.3	27.2	23.7
004/TauUncJ	IF	22.0	24.8	21.0	18.2	27.3	29.6	28.6	32.3
005/TauUncJ	AL	26.1	22.6	22.0	16.2	28.2	27.0	29.4	27.6
005/TauUncJ	IF	23.7	24.5	17.5	21.8	27.0	28.1	28.7	29.6
006/TauUncJ	AL	13.3	17.4	8.6	12.9	18.6	23.4	27.2	25.9
006/TauUncJ	IF	15.2	23.5	9.8	11.8	20.8	28.0	26.2	31.0
018/UncJ	AL	23.0	24.9	18.7	22.4	26.4	29.0	29.4	29.6
018/UncJ	IF	21.0	22.6	16.3	19.5	24.7	24.9	29.4	26.2
019/TauUncJ	AL	23.0	25.5	20.6	22.8	27.0	27.9	29.6	31.1
019/TauUncJ	IF	24.7	25.7	21.7	22.9	26.6	27.8	30.1	30.8
032/GeniUncJ	AL	22.9	26.5	18.4	23.9	25.4	29.7	28.6	33.1
032/GeniUncJ	IF	24.6	30.2	20.5	27.2	25.9	31.4	29.3	33.6
040/TauUncJ	AL	19.8	19.3	14.2	14.4	26.1	24.0	26.7	26.8
040/TauUncJ	IF	21.9	24.6	16.5	22.2	23.6	26.7	27.8	30.4
041/TauUncJ	AL	14.9	17.6	11.3	13.1	18.5	19.1	20.0	24.2
041/TauUncJ	IF	15.9	20.0	11.9	13.3	21.2	23.3	22.0	24.7
061/GeniUncJ	AL	24.6	27.2	20.3	19.9	27.3	28.3	29.3	31.4
061/GeniUncJ	IF	22.9	29.0	20.3	21.0	25.8	32.1	26.2	33.6

Supplemental Table S3: Pairwise comparison of strain effects on lifespan.
Significance (p-value) of all pairwise comparisons (1df) between strain groups from log rank tests. Overall (9df) significance p<2.2e-16.

	003/ UncJ	004/ TauUncJ	005/ TauUncJ	006/ TauUncJ	018/ UncJ	019/ TauUncJ	032/ GeniUncJ	040/ TauUncJ	041/ TauUncJ
004/TauUncJ	2.8e-07								
005/TauUncJ	2.1e-07	0.7686							
006/TauUncJ	2.5e-11	0.0167	0.0026						
018/UncJ	9.2e-08	0.9646	0.9323	0.0087					
019/TauUncJ	0.00055	0.0486	0.0802	2.7e-05	0.0466				
032/GeniUncJ	0.04860	0.0022	0.0022	2.8e-07	0.0011	0.1588			
040/TauUncJ	1.3e-10	0.2886	0.0957	0.1159	0.2707	0.0016	2.8e-05		
041/TauUncJ	<2e-16	2.8e-09	5.6e-15	0.0319	3.2e-11	<2e-16	<2e-16	2.9e-08	
061/GeniUncJ	0.02113	0.0085	0.0125	1.4e-06	0.0085	0.3662	0.69	0.00012	<2e-16

Supplemental Table S4: Diet effects on mean lifespan by sex and strain

Strain	Sex	Ratio [IF/AL] (95% CI)	p
003/UncJ	Female	0.55 (0.28, 1.06)	0.074
003/UncJ	Male	0.74 (0.39, 1.42)	0.367
004/TauUncJ	Female	1.03 (0.55, 1.94)	0.915
004/TauUncJ	Male	0.32 (0.16, 0.63)	9e-04
005/TauUncJ	Female	1.62 (0.84, 3.12)	0.149
005/TauUncJ	Male	0.46 (0.24, 0.91)	0.026
006/TauUncJ	Female	0.81 (0.43, 1.53)	0.518
006/TauUncJ	Male	0.69 (0.35, 1.36)	0.284
018/UncJ	Female	1.32 (0.7, 2.49)	0.390
018/UncJ	Male	1.9 (0.97, 3.72)	0.059
019/TauUncJ	Female	0.8 (0.43, 1.5)	0.485
019/TauUncJ	Male	1.09 (0.58, 2.05)	0.788
032/GeniUncJ	Female	0.71 (0.38, 1.34)	0.294
032/GeniUncJ	Male	0.66 (0.34, 1.28)	0.222
040/TauUncJ	Female	1.09 (0.58, 2.05)	0.797
040/TauUncJ	Male	0.41 (0.21, 0.8)	0.009
041/TauUncJ	Female	0.65 (0.34, 1.23)	0.183
041/TauUncJ	Male	0.59 (0.31, 1.15)	0.122
061/GeniUncJ	Female	1.04 (0.53, 2.07)	0.900
061/GeniUncJ	Male	0.56 (0.28, 1.11)	0.095

Supplementary Table S5: Coefficient of variation (CV) in lifespan by diet, strain, and sex

Strain	Diet	CV_Female	CV_Male
003/UncJ	AL	0.19	0.13
004/TauUncJ	AL	0.25	0.27
005/TauUncJ	AL	0.19	0.28
006/TauUncJ	AL	0.47	0.40
018/UncJ	AL	0.24	0.21
019/TauUncJ	AL	0.25	0.18
032/GeniUncJ	AL	0.26	0.24
040/TauUncJ	AL	0.29	0.28
041/TauUncJ	AL	0.26	0.30
061/GeniUncJ	AL	0.25	0.19
003/UncJ	IF	0.33	0.23
004/TauUncJ	IF	0.24	0.28
005/TauUncJ	IF	0.22	0.17
006/TauUncJ	IF	0.44	0.37
018/UncJ	IF	0.28	0.20
019/TauUncJ	IF	0.17	0.18
032/GeniUncJ	IF	0.24	0.15
040/TauUncJ	IF	0.32	0.13
041/TauUncJ	IF	0.33	0.29
061/GeniUncJ	IF	0.16	0.20

Supplementary Table S6: Trait definitions.
Filename: Tab_S6.csv
Content: Description of study data.
Contains the following fields:
Task Name: corresponds to 'task_name' in dataset
Task Description: defines 'task_name'
Trait Name: corresponds to 'variable' in dataset (phenotypic trait)
Trait Description: defines 'variable'

Supplemental Table S7: Phenotyping domains with age ranges.
Due to scheduling constraints, it was not possible to test all mice at precisely the same ages. In this table we record for each phenotyping event (indexed by phenotyping domain and timepoint [in months]) the youngest (min_age), median (median_age), and oldest (max_age) ages at which mice were tested.

Domain	Timepoint	min_age	med_age	max_age
CBC	10.00	9.20	11.40	12.10
CBC	22.00	21.40	23.40	24.20
CBC	34.00	33.60	34.70	35.80
FLOW	5.00	3.30	5.20	5.90
FLOW	16.00	14.30	16.30	17.60
FLOW	28.00	26.20	28.20	28.80
Fasted.GI	6.00	3.70	5.60	6.30
Fasted.GI	17.00	14.70	16.70	17.20
Fasted.GI	28.00	17.30	28.70	29.70
Frailty	5.00	3.70	5.00	7.40
Frailty	10.00	7.90	10.10	10.90
Frailty	16.00	14.10	16.10	16.70
Frailty	22.00	20.80	22.00	23.20
Frailty	28.00	26.10	28.10	28.80
Frailty	33.00	32.10	33.50	34.10
NMR	10.00	8.40	10.60	11.40
NMR	23.00	20.60	22.50	23.20
NMR	32.00	32.60	33.60	34.50

Supplementary Table S8: Diet effect in recombinant inbred strain panel.
Filename: Tab_S8.csv
Content: Longitudinal analysis test statistics for diet effect on traits. File contains the following fields:
Variable: [trait domain]_[trait name].
Contrast: Direction of effect estimated
Timepoint: Timepoint in weeks. Not applicable for traits summarized at the individual level described in Methods: Summarization of trajectories.
Estimate, Estimate_Male, Estimate_Female: estimate of adjusted diet association, as described in Methods: Estimation of population-averaged intervention effect. Adjustment factors as described in Methods: Modeling strategy.
p.value, p.value_Female, p.value_Male: p-value for estimates. Adjustment factors as described in Methods: Modeling strategy.
q.value, q.value_Female, q.value_Male: false discovery rate adjusted significance.

Supplementary Table S9: Diet effect in recombinant inbred strain panel.
Filename: Tab_S9.csv
Content: Estimation of gene-by-treatment interaction as described in Methods: "Estimation of GxT from random effects."
Variable: [trait domain]_[trait name].
p.value_sd_diet: p-value for diet effect heterogeneity across strains.
q.value_sd_diet: false discovery rate adjusted of above.
Coefficient_[strain]: diet random effect, corresponding to the deviation from fixed effect.

Supplementary Table S10: Strain and diet random effect correlation.
Filename: Tab_S10.csv
Content: Estimation of correlation between strain and diet random effects as described in Methods: "Estimation of GxT from random effects."
Variable: [trait domain]_[trait name].
cor_diet_strain_res: correlation between strain and diet random effects.

Supplementary Table S11: Diet effect in outbred population.
Filename: Tab_S11.csv
Content: Longitudinal analysis test statistics for diet effect on traits in outbred population. File contains the following fields:
Variable: Trait name
Contrast: Direction of effect estimated
Timepoint: Timepoint in years. Not applicable for traits summarized at the individual level described in Methods: Summarization of trajectories.
Estimate: Estimate of adjusted diet association, as described in Methods: Comparative analysis. Note female-specific, per study design. A rank-normalized effect sizes (RNES)—defined as the estimated marginal mean difference on the rank-z transformed scale.
p.value: p-value for estimates. Adjustment factors as described in Methods: Modeling strategy.
q.value: false discovery rate adjusted significance.

Supplementary Table S12: Phenotype correlations with lifespan.
Filename: Tab_S12.csv
Content: Lifespan association test statistics as described in Methods: "Trait Association with Lifespan."
File contains the following fields:
Domain: Type of assay. Variable: Trait name
Timepoint: Timepoint in weeks.
p_adj: p-value of trait-lifespan association adjusted for sex, diet, strain, and body weight.
q_adj: false discovery rate adjusted of above.
b_adj: adjusted (partial) correlation of lifespan with trait.

Supplementary Methods

Quantification of trajectories. For some outcomes it was possible summarize y at the individual mouse level. Body-weight trajectories were summarized per mouse as mean mass (MM) and total AUC (+AUC); +AUC was computed with the trapezoidal rule via the integrate.xy() function of the sfsmisc R package (v.1.1-16). Per-mouse frailty trajectories were summarized as linear coefficients (slopes) on PLL scale to describe pace of aging, and final FI score was used as a proxy for lifetime multisystem frailty accumulation. Per-mouse temperature trajectories were likewise summarized as linear coefficients (slopes) on proportion of life lived (PLL [scaled age]). For these phenotypes, a simplified model was specified without fixed effects for timepoint, random effects for Id, or random effects for collection date (batch).

Estimation of population-averaged intervention effect. For the models described above, corresponding p-values for diet fixed effect overall and within sex (and timepoint, where modeled) were computed via contrasts of model-based means estimated via the emmeans package in R. Whereas stratifying can lead to loss of information and reduced statistical power, applying pairwise tests within a single model framework provides more accurate and reliable comparisons by accounting for the overall data structure. To control for the false discovery rate across multiple phenotypes, we applied the Benjamini-Hochberg (BH) procedure to adjust diet p-values.

Estimation of GxT from random effects. To estimate how genetic background interacts with dietary treatment (GxT interaction), we tested for diet effect heterogeneity across strains via the ranova function from the lmerTest package in R. Correlation between random effect for strain and random diet effect within strain (IF v AL), denoted as "ρ", indicated how strain-specific diet effect varied across strain-specific estimates for baseline control. To control for the false discovery rate, we applied the Benjamini-Hochberg (BH) procedure to adjust p-values for diet random effect and, separately, ρ across multiple phenotypes.

Mouse model selection. We applied random effects post-estimation to the panel of mouse strains as models for heterogeneous responses to intermittent fasting. Best linear unbiased predictions (BLUPs) provide empirical Bayes (EB) estimates of random effects, ζ_{EB}, offering a principled way to quantify strain-level deviations in IF response from population mean response while accounting for uncertainty and shrinkage toward the overall mean. In this context, ζ_{EB} capture the magnitude and direction of each strain's response to intermittent fasting (IF) conditional on the fitted model structure. To generate interpretable strain-specific diet effect scores, we summed ζ_{EB} and population mean diet response computed from post-estimated model contrasts taking sex-by-diet interaction into account. For hematologic and immunologic phenotypes, total strain-specific diet effects scaled within strain were grouped into clusters via unsupervised learning and co-displayed with heatmaps organized by health outcome and strain generated via the tidyHeatmap package in R, revealing groups of strains with distinctive treatment response.

Comparative analysis - Trait selection. Reanalysis focused on a shared subset of trait domains: body weight, body composition, frailty, hematology, and immunology. Hematologic and immunologic traits were selected based on their prominence in the original DRiDO publication. Comparability across the two studies for body composition analysis and immunophenotyping is lesser than for other phenotypic domains as scientific staff implemented new assessment modalities in the period between conducting DRiDO and the CC, transitioning from dual-energy x-ray absorptiometry (DEXA) to nuclear magnetic resonance body composition assessment EchoMRI-3-in-1 Whole Body Resonance Analyzer and from fluorescence-activated cell sorting (FACS) to flow cytometry-based immunophenotyping.

Supplementary Discussion

Sexual dimorphism in longevity intervention response. Sexual dimorphism of genotype effects on phenotypic traits has been observed in large-scale mouse studies such as the International Mouse Phenotyping Consortium (IMPC) database (>14k wildtype animals and >40k mutant mice) [1] and the Intervention Testing Program [2, 3]. The latter also revealed unexpected sex differences in geroprotective effects of some interventions (e.g., [4, 5]). In keeping with our findings, dietary treatments have been shown to affect lifespan differently in males and females [6–8]. These findings may reflect sex-specific dietary requirements for healthy aging that could not be explored in our parallel study of female outbred mice.

Lifespan response to IF differs between inbred and outbred mice. We previously reported survival data from the DRiDO study for female Diversity Outbred (DO) mice including AL and 2-day IF cohorts [9]. The DO are derived from the same founders as the CC strains and thus share the same genetic variants. In addition, the two studies were carried out concurrently in the same mouse facilities. In the DRiDO study, which included only female mice, the 2-day IF intervention led to significant lifespan extension – population-level median lifespan increased by months. We further observed that lifespan increase was more pronounced in mice with lower pre-intervention body weight and no extension of lifespan was observed for the heaviest mice. In the CC study, aggregate lifespan data revealed sexually dimorphic lifespan response to IF. We observed significant lifespan extension in aggregated (across-strain) data for male mice only, which is discordant from the result in outbred mice.

Heritability of lifespan is concordant in inbred CC and outbred DO mice. It is common in preclinical studies to test intervention effects on a single inbred mouse strain, typically C57BL/6. Yet lifespan is a complex trait that is influenced by a large number of genetic and non-genetic factors that drive phenotype heterogeneity. Estimates of heritability provide some information on the relative importance of genetic factors in comparison with non-shared environmental factors. In both the CC Longitudinal Study and DRiDO, we imposed precise control over the environment and husbandry of mice to minimize environmental variance and selected mouse models with exceptionally high levels of diversity to maximize the genetic contribution thereby creating the most favorable setting for high heritability of lifespan and other phenotypes. As in DRiDO, we found genetic background proved to be the more important factor in determining lifespan. Heritability of lifespan in the CC study was nearly completely accounted for by additive genetic variance—ie., variance due to mean effects of single alleles. We estimated the narrow and broad heritabilities for lifespan (adjusted for sex and diet) as being nearly equal. This does not preclude the possibility of gene interactions affecting lifespan and IF response as traits with large additive variance can be driven by physiological epistasis [10].

Genetics alter phenotypic response to intermittent fasting. We identified gene-by-treatment interaction across metabolic, hematologic, and immunologic profiles. Shifts from strain-normative phenotype were often distinguishable by both genetic strain and sex. Intervention studies that incorporate genetic variability will be important for success

in translational metabolic research. The Collaborative Cross (CC) study presented here demonstrates that dietary intervention response is nonuniform and patterned across genetic backgrounds. By profiling intermittent fasting effects across 10 inbred strains and multiple physiological systems, this study establishes a reusable library of mouse models for mechanistic follow-up, enabling validation of genetically driven heterogeneity in intervention outcomes previously observed among outbred DO mice. Future studies using these mouse models could regenerate the same genotypes to replicate our findings as well as exploring new genetic backgrounds, including new CC strains and their F1 hybrid to further explore the variability in dietary intervention outcomes.

Concordance and divergence in the impacts of intermittent fasting on health among genetically diverse inbred and outbred mice. The Collaborative Cross (CC) Longitudinal Study, which explores gene-by-treatment interaction in a panel of recombinant inbred strains, was conducted in parallel with DRiDO, a study of dietary restriction (including 2-day IF) in female DO mice. Uniquely, the two studies were conducted in the same laboratory facility during the same time period. IF produced broadly concordant effects on health across genetically diverse mouse populations, with some notable divergences. "Intermittent fasting has received attention as an alternative lifespan extending intervention [11]. We elected to implement a weekly schedule with 2 consecutive days of fasting in the CC study to ensure comparability with our DRiDO study. The relative absence of lifespan response in CC mice, compared to DO mice, was a surprising outcome that limited our ability to identify responder and non-responder CC strains. In contrast, many of the metabolic and blood phenotypes displayed a robust response to IF that enabled us to demonstrate genetic heterogeneity and attribute distinct response patterns to specific CC strains."

In both the outbred (DRiDO) and inbred (CC) studies, IF significantly reduced lifetime body mass in females, though strain-specific deviations in late-life weight trajectories were observed among certain inbred lines. Lean mass was preserved in outbred females on IF, contrasting with reductions seen across the recombinant inbred strains. IF elevated red cell distribution width (RDW-CV) across both cohorts, highlighting a robust, genotype-independent biomarker response warranting mechanistic investigation, especially in light of consistently strong predictive utility as a longevity biomarker (see Results: Longevity Biomarkers). Leveraging both outbred and recombinant inbred mouse populations provides complementary strengths for dissecting the biology of dietary interventions. Outbred populations, like those used in the DRiDO study, are powerful for genetic mapping and broadly representative of genetic diversity, a strategy increasingly adopted across model organisms since its formalization in the early 2000s [12]. In contrast, the recombinant inbred panel screen used here—less commonly employed—offers a replicable framework for identifying strain-specific responses.

Inflection point in thermoregulatory response. A rapid decline in body temperature (TB) is an indicator of immanent death in standard laboratory mice (e.g., C57BL/6) [13]. The predictive power of TB was recently confirmed in DO mice [14]. In the present study we sampled TB at 5 timepoints and rescaled chronological age to proportion of life lived. We confirmed the expected decline in TB with age, and observed an inflection point at 80% of life lived (80 PLL) that

was most pronounced in strains CC004, CC006, CC019, and CC041. The non-linear rate of change as a function of PLL was not impacted by IF. These trends are apparent at the strain-average level, but at the individual level can be obscured by noise (measurement error) or limited time density of sampling (6-month intervals). These finding support TB as a biomarker of aging and predictor of lifespan that is consistent across genetic background.

Comprehensive longitudinal phenotyping identifies longevity biomarkers. In our previous work [9], health and metabolic traits were sensitive to diet but were poor predictors of lifespan, while hematologic and immune traits showed strong dietary response and lifespan prediction. That is, counterintuitively, variability in lifespan within treatment group was not explainable by favorable changes in response to dietary restriction. We looked for replications of these patterns in the CC, considering each phenotypic domain and its relevance as a marker for longevity. As in DRiDO, the current study found that IF-induced phenotype changes in health and metabolic traits did not necessarily translate into lifespan extension. In light of these contradictory effects, and the adoption of fasting by people who seek to improve health and increase lifespan, further study of health effects of IF should be a priority for future research.

A recent trial [15] found that despite improved human adherence to IF resulting in significant body weight and fat loss, clinically meaningful improvements in systemic inflammatory markers or glucose-insulin metabolism were not observed. These findings further validate that weight reduction does not always translate into improved metabolic heath and highlights a priority area for future research on IF as an anti-aging intervention.

Computational methods for recombinant inbred strain panel screens. Recombinant inbred panel screens, such as the one presented here, require large sample sizes to achieve sufficient statistical power for detecting disaggregated strain-specific effects. This scale of experimentation is often impractical. Computational methods can be leveraged to maximize power to detect variability in strain-specific intervention response. In this study, we introduced a novel application of empirical Bayes estimation of strain-specific random effects to identify mouse models exhibiting heterogeneous responses to intervention. This approach conceptualizes strain in the panel screen as samples drawn from a broader population, enabling genotype-by-treatment (GxT) interactions to be modeled as a single variance component representing intervention effect variability across genetic backgrounds. The magnitude of this variance component can be compared across models of standardized phenotypes to identify traits more pronounced GxT interaction effects. Further, by leveraging empirical Bayes estimates, distinctive strains can be identified by examining the extreme values of the best linear unbiased predictors (BLUPs) for strain-specific intervention effects. E.g., selecting strains with maximum and minimum BLUPs for dietary effect enables the selection of strain pairs that exhibit divergent, and potentially directionally opposite, responses to the intervention.

Limitations This study aimed to investigate heterogeneity of lifespan extension in response to IF across a panel of 10 recombinant inbred strains. However, the observed effect of IF on lifespan was modest relative to the within-strain variance, limiting our ability to establish strong conclusions about strain-specific differences in response. Future studies may benefit from examining genotype-by-treatment (GxT) interactions using dietary interventions with more pro-

nounced effects on lifespan—such as 40% caloric restriction—though IF remains of high interest due to its tolerability [16].

Another limitation lies in the study's sample composition. The genetic composition of most CC strains equally represents contributions from the eight founders with no obvious allelic distortion at a per locus basis. In this sense any selection of strains would be representative of the population of (existing and potential) CC strains. However, availability of CC strains was contingent on successful breeding in the repository colony at the time of this study. Thus, we cannot exclude the possibility that theses 10 strains may be atypical of the larger CC strain set in some regards.

Future studies with more densely sampled time series data will avoid data sparsity issues across the full range of PLL. We were able to demonstrate this method for temperature, which was more frequently collected than other phenotypes (5 measurements vs. 2 to 3). Normalizing chronological age across all phenotypes could reveal non-linear dynamics in IF response. Such studies are in the pipeline.

Despite these limitations, the study design proved effective in detecting robust treatment responses in other phenotypes. We observed significant and substantial heterogeneity in body weight and composition responses to IF, suggesting that the design is well-suited for outcomes with stronger intervention effects. These findings highlight the importance of aligning study design with the expected magnitude of treatment effects when investigating complex traits across genetically diverse populations.

References

1. Karp, N. A. *et al.* Prevalence of sexual dimorphism in mammalian phenotypic traits. *Nature Communications* **8,** 15475 (2017).

2. Nadon, N. L., Strong, R., Miller, R. A. & Harrison, D. E. NIA Interventions Testing Program: investigating putative aging intervention agents in a genetically heterogeneous mouse model. *EBioMedicine* **21,** 3–4 (2017).

3. Austad, S. N. Sex differences in health and aging: a dialog between the brain and gonad? *Geroscience* **41,** 267–273 (2019).

4. Miller, R. A. *et al.* Rapamycin-mediated lifespan increase in mice is dose and sex dependent and metabolically distinct from dietary restriction. *Aging Cell* **13,** 468–477 (2014).

5. Harrison, D. E. *et al.* Acarbose, 17-α-estradiol, and nordihydroguaiaretic acid extend mouse lifespan preferentially in males. *Aging Cell* **13,** 273–282 (2014).

6. Liao, C.-Y., Rikke, B. A., Johnson, T. E., Diaz, V. & Nelson, J. F. Genetic variation in the murine lifespan response to dietary restriction: from life extension to life shortening. *Aging Cell* **9,** 92–95 (2010).

7. Cheng, C. J., Gelfond, J. A., Strong, R. & Nelson, J. F. Genetically heterogeneous mice exhibit a female survival advantage that is age-and site-specific: Results from a large multi-site study. *Aging Cell* **18,** e12905 (2019).

8. Unnikrishnan, A. *et al.* Reevaluation of the effect of dietary restriction on different recombinant inbred lines of male and female mice. *Aging Cell* **20,** e13500 (2021).

9. Di Francesco, A. *et al.* Dietary restriction impacts health and lifespan of genetically diverse mice. *Nature* **634,** 684–692 (2024).

10. Cheverud, J. M. & Routman, E. J. Epistasis and its contribution to genetic variance components. *Genetics* **139,** 1455–1461 (1995).

11. Longo, V. D. & Mattson, M. P. Fasting: Molecular mechanisms and clinical applications. *Cell Metabolism* **19,** 181–192 (2014).

12. De Koning, D.-J. & McIntyre, L. M. Back to the future: multiparent populations provide the key to unlocking the genetic basis of complex traits. *G3: Genes, Genomes, Genetics* **7,** 1617–1618 (2017).

13. Ray, M. A., Johnston, N. A., Verhulst, S., Trammell, R. A. & Toth, L. A. Identification of markers for imminent death in mice used in longevity and aging research. *Journal of the American Association for Laboratory Animal Science* **49,** 282–288 (2010).

14. Luciano, A. *et al.* Longitudinal fragility phenotyping contributes to the prediction of lifespan and age-associated morbidity in C57BL/6 and Diversity Outbred mice. *GeroScience* **46,** 4937–4954 (2024).

15. Tosti, V. *et al.* When a calorie is not a calorie: Metabolic and molecular effects of intermittent fasting in humans; Exploratory outcomes of a randomized clinical trial. *Aging Biol* **1,** 20230013 (2023).

16. Longo, V. D. & Panda, S. Fasting, circadian rhythms, and time-restricted feeding in healthy lifespan. *Cell Metabolism* **23,** 1048–1059 (2016).
